# Complex Class 1 Integron in a Clinical *Escherichia coli* Strain From Vietnam Carrying Both *mcr-1* and *bla*_NDM–1_

**DOI:** 10.3389/fmicb.2019.02472

**Published:** 2019-10-31

**Authors:** Hong-Ngoc Le-Vo, Phuong Thi-Bich Tran, Lien Le, Yuki Matsumoto, Daisuke Motooka, Shota Nakamura, James W. Jones, Tetsuya Iida, Van Cao

**Affiliations:** ^1^Department of Immunology and Microbiology, Pasteur Institute in Ho Chi Minh City, Ho Chi Minh City, Vietnam; ^2^Genome Information Research Center, Research Institute for Microbial Diseases, Osaka University, Osaka, Japan; ^3^Department of Bacterial and Parasitic Diseases, Armed Forces Research Institute of Medical Sciences, Bangkok, Thailand; ^4^Department of Bacterial Infections, Research Institute for Microbial Diseases, Osaka University, Osaka, Japan

**Keywords:** *mcr-1*, *bla*_NDM–1_, colistin, carbapenemase, complex class 1 integron, *sul3*-type integron, *Escherichia coli*

## Abstract

The co-production of MCR and carbapenemase in *Enterobacteriaceae* has been previously reported. Here, we describe a clinical strain of *Escherichia coli* from Vietnam carrying both *mcr-1* and *bla*_NDM–1_. Whole-genome sequencing showed that the genome of this strain consists of a 4,975,832-bp chromosome and four plasmids. The *mcr-1* and *bla*_NDM–1_ genes are located on IncI2 and IncA/C2-type plasmids, respectively. Genetic analysis revealed the presence of a multidrug-resistant region with the structure of a novel complex class 1 integron including a class 1 integron region bearing two 5′ conserved segments and one 3′ conserved segment and two complete structures of IS*CR1*. The complex integron contains aminoglycoside resistance genes *aadA2*, *aadB*, *strA*, *strB*, and *aphA6*, quinolone resistance gene *qnrA1*, extended-spectrum β-lactamase gene *bla*_OXA–__4_, and a Tn*125*-like transposon bearing *bla*_NDM–1_. In addition, the *dfrA12*-*gcuF*-*aadA2*-*cmlA1*-*aadA1-qacH* gene cassette array belonging to the *sul3*-type integron was also identified, but the region found downstream of the gene cassette array is the IS*440*-*tet(M)*-IS*26* element instead of the *sul3* gene. The results further support that *Enterobacteriaceae* isolates co-harboring *mcr* and *bla*_NDM_ are widely being distributed. The structural characteristics of the complex integron reveal that IS*CR1* elements play an important role in the mobilization of *bla*_NDM–1_ and the development of multidrug-resistant regions.

## Introduction

Infections caused by carbapenem-resistant *Enterobacteriaceae* (CRE) are a serious problem for global health. The main mechanism for carbapenem resistance in bacteria is the production of carbapenemases, out of which New Delhi metallo-lactamase (NDM) is one of the most common groups (Iovleva and Doi, 2017). The *bla*_NDM–1_ gene was mainly found on multiple plasmid types, including IncA/C, IncFIA, IncFIB, IncFII, and IncX3 (Wu et al., 2019). This indicates that the horizontal gene transfer mediated by plasmids plays a vital role in the widespread dissemination of *bla*_NDM–1_. Infections caused by NDM-1-producing *Enterobacteriaceae* are of major concern because those isolates tend to spread among patients, which poses a challenge to control the outbreak of CRE carrying *bla*_NDM–1_. In addition, NDM-1-producing plasmids also carry additional genes conferring resistance to other antibiotics, such as aminoglycosides (*aacA29* and *aadA16*), chloramphenicol (*catA1* and *cmlA1*), and tetracyclines (*tetB*) (Rojas et al., 2016), resulting in the limited choices of antibiotics in treatment. In several plasmids, the *bla*_NDM–1_ gene was flanked by two copies of insertion sequences (e.g., IS*Aba125*, IS*26*, and IS*3000*), which generate the composite transposons carrying *bla*_NDM–1_ (Wu et al., 2019). Class 1 integrons were found linked to the complete structures of insertion sequence common region 1 (IS*CR1*) including a copy of IS*CR1*, a variable region (VR) bearing resistance genes, and a copy of the 3′ conserved segment (CS) of the class 1 integron to construct large genetic elements that are usually called “complex class 1 integrons” (Toleman et al., 2006). Some reports showed that *bla*_NDM–1_ was also inserted into the IS*CR1*-linked VR of complex class 1 integrons (Chen et al., 2014; Li et al., 2014), suggesting that the mobilization of *bla*_NDM–1_ is most likely associated with IS*CR1* as well as the formation of composite transposons.

Colistin is one of the few antibiotics that are still effective against CRE. However, the mutation of chromosomal genes involved in lipopolysaccharide production have impacts on the susceptibility of bacteria to colistin (Olaitan et al., 2014). Moreover, colistin resistance has become more serious since plasmid-mediated colistin resistance gene *mcr-1* was first reported in 2015 (Liu et al., 2016). Presently, eight additional *mcr* genes (*mcr-2* to *mcr-9*) and their variants have been described in *Enterobacteriaceae* (Xavier et al., 2016; AbuOun et al., 2017; Borowiak et al., 2017; Carattoli et al., 2017; Yin et al., 2017; Wang et al., 2018c; Yang et al., 2018; Carroll et al., 2019). Although the presence of *mcr* was found on chromosomes (Zhou et al., 2017), the *mcr* genes were mainly reported to be carried on different plasmid types, such as IncI2, IncHI2, IncX4, IncP, and IncFII (Xavier et al., 2016; Matamoros et al., 2017; Wang et al., 2018c). IncX4, IncI2, and IncHI2 are the most common types of plasmids carrying *mcr-1* (Matamoros et al., 2017). On the other hand, these plasmids also harbor additional resistance genes (e.g., *bla*_CTX__–__M__–__14_, *fosA3*, *floR*, and *oqxAB*) and can be transferred via conjugation (Wu et al., 2018). However, the IS*Apl1* composite transposon was considered as the main vehicle for the widespread distribution of *mcr-1* (Wang et al., 2018a).

The *mcr-1* gene was detected in colistin-resistant *Enterobacteriaceae* isolated from clinical samples (Du et al., 2016; Tada et al., 2017) and has been commonly found in colistin-resistant *Escherichia coli* recovered from farm animals and foods in China (Wang et al., 2018b; Liu et al., 2019) and Vietnam (Trung et al., 2017; Yamaguchi et al., 2018). More importantly, a recent study showed that the existence of *mcr-1* is the predominant genotype of colistin-resistant *E. coli* isolated from healthy residents in Vietnam (Yamamoto et al., 2019). The prevalence of *mcr-1* in multiple sources revealed that these sources might be potential reservoirs for the wide spread of colistin-resistant bacteria producing MCR-1 in livestock-producing countries such as Vietnam. Significantly, clinical *mcr-1*- and *bla*_NDM_-positive *Enterobacteriaceae* isolates, particularly *E. coli* and *Klebsiella pneumoniae*, were recently found in various regions (Du et al., 2016; Mediavilla et al., 2016; Zheng et al., 2016), which poses a threat to the effectiveness of carbapenems and colistin. In Vietnam, *bla*_NDM_ is the most common genotype reported in carbapenemase-producing *E. coli* (Malchione et al., 2019), and *mcr-1* has been frequently found in colistin-resistant *E. coli* isolated from different sources (Trung et al., 2017; Yamaguchi et al., 2018). Thus, we hypothesized that multidrug-resistant *Enterobacteriaceae* strains that simultaneously harbor *mcr-1* and *bla*_NDM_ are currently circulating in Vietnam. Recently, we have obtained a clinical *E. coli* strain resistant to multiple antibiotics, including carbapenems and colistin. The aim of this study was to elucidate the antibiotic resistance mechanisms of the isolate, especially to detect the coexistence of *mcr-1* and *bla*_NDM_.

## Materials and Methods

### Bacterial Isolate

*Escherichia coli* EC17GD31 was recovered from the blood sample of a patient admitted to the intensive care unit of Nhan Dan Gia Dinh hospital (Ho Chi Minh City, Vietnam) in March 2017. The patient was diagnosed as having acute respiratory distress syndrome, pneumonia, and acute kidney injury and had a medical history of cerebral infarction. EC17GD31 was isolated in the routine diagnostic laboratory of the hospital and then sent to Pasteur Institute in Ho Chi Minh City for further analysis. The isolate was grown on brain heart infusion agar overnight at 37°C and species-identified by using VITEK 2 system (BioMérieux, United States).

This study received ethical approval from the Institutional Ethics Committee in Biomedical Research, Pasteur Institute in Ho Chi Minh City. Informed consent was not required for this study, since the strain was isolated as a part of the routine hospital laboratory procedures.

### Antibiotic Susceptibility

The minimum inhibitory concentrations (MICs) of amikacin, gentamicin, ampicillin, amoxicillin-clavulanic acid, cefotaxime, ceftazidime, ceftriaxone, cefepime, ertapenem, imipenem, meropenem, cefoxitin, ciprofloxacin, chloramphenicol, tetracycline, and trimethoprim-sulfamethoxazole were determined by agar dilution method, although MICs of colistin and tigecycline were performed with broth microdilution. Colistin and tigecycline resistance was interpreted according to the breakpoints of the European Committee on Antimicrobial Susceptibility Testing (EUCAST^1^), whereas other antibiotics were identified according to the guidelines of the Clinical and Laboratory Standards Institute (CLSI, 2018).

### Conjugation Assay

The transferability of the *mcr-1*-bearing plasmid was examined by conjugation experiment. *E. coli* J53, which is resistant to sodium azide, was used as the recipient. *E. coli* J53 transconjugants that harbor MCR-1-encoding plasmids were selected on brain heart infusion agar plates containing 100 μg/ml sodium azide and 2 μg/ml colistin. The presence of *mcr-1* in the transconjugants was confirmed by PCR as previously described (Liu et al., 2016).

### Whole-Genome Sequencing and Bioinformatics

The genomic DNA was extracted from EC17GD31 using a QIAamp DNA Mini Kit (Qiagen, Germany). Whole-genome sequencing was carried out using the MinION sequencer (Oxford Nanopore Technologies, United Kingdom). Genome assembly was performed by Canu 1.6 (Koren et al., 2017) and then corrected using Pilon 1.22 (Walker et al., 2014) with reads obtained from 250-bp paired-end Illumina MiSeq sequencing (Illumina Inc., United States). NCBI Prokaryotic Genome Annotation Pipeline^2^ was used to annotate the genomic sequences. Multilocus sequence typing of the isolate, plasmid replicon typing, and identification of resistance genes were done using tools from the Center for Genomic Epidemiology website^3^. Insertion sequences were identified based on the ISfinder database^4^. Sequence comparison was completed using BLASTn^5^. The circular image and linear comparison figures of sequences were generated by CGView (Stothard and Wishart, 2004) and Easyfig (Sullivan et al., 2011), respectively.

### Phylogenetic Analysis of IncI2 Plasmids

Complete sequences of IncI2 plasmids carrying *mcr-1* (*n* = 21) were obtained from GenBank (Supplementary Table 1). A sum of 35 genes were identified as the core genome of the plasmids (including pGD31-MCR) using OrthoFinder version 2.3.3 (Emms and Kelly, 2015). The alleles of these genes were aligned as protein sequences using MAFFT version 7.427 (Katoh and Standley, 2013), and then the protein sequences of each plasmid were concatenated into a single sequence. RAxML version 8.2.12 (Stamatakis, 2014) was used to infer a phylogenetic tree with a 1000-bootstrap test. The phylogenetic tree was drawn using the iTOL web server (Letunic and Bork, 2019).

### Nucleotide Sequence Accession Numbers

Complete sequences of the chromosome and plasmids of *E. coli* EC17GD31 have been deposited in GenBank under the accession numbers CP031293 to CP031297.

## Results and Discussion

### Genomic Characteristics of *E. coli* EC17GD31

The genome of *E. coli* EC17GD31 consists of a 4,975,832 bp chromosome with an average 50.46% GC content and four circular plasmids (Table 1). The isolate was resistant to all tested antibiotics, except for tigecycline (Table 2). It is notable that EC17GD31 was resistant to both carbapenems (MICs ≥ 8 μg/ml) and colistin (MIC = 4 μg/ml). Identification of 22 antibiotic resistance genes showed good correlation to the antibiotic susceptibility pattern of *E. coli* EC17GD31 (Table 1). The detection of *bla*_TEM–__1__B_, *bla*_CTX–M–__55_, *bla*_OXA–__4_, and *bla*_NDM–1_ explained the resistance to all tested β-lactam antibiotics, including extended-spectrum cephalosporins and carbapenems (Table 2). The existence of *mcr-1* and efflux pump-encoding genes (*cmlA1* and *floR*) was related to the resistance to colistin and chloramphenicol, respectively. In addition, the genome of EC17GD31 carries genes conferring the resistance to aminoglycosides (*aadA1*, *aadA2*, *aadB*, *aph(3*′*)-Ia*, *aphA6*, *strA*, and *strB*), quinolones (*qnrA1*), tetracyclines [*tet(A)* and *tet(M)*], sulfonamides (*sul1*, *sul2*, and *sul3*), and trimethoprim (*dfrA12*) (Table 1). The multidrug resistance transporter-encoding gene *mdf(A)* was also found in EC17GD31, which probably contributed to the resistance of this isolate to tetracycline, chloramphenicol, and ciprofloxacin (Edgar and Bibi, 1997). The *bla*_CTX–M–__55_ encoding extended-spectrum beta-lactamase (ESBL) and *mdf(A)* genes are located on the chromosome, whereas other resistance genes are present on three plasmids. Moreover, three mutations in type II topoisomerases were identified, including substitution Ser83Leu and Asp87Tyr in GyrA and Ser80Ile in ParC. These mutations are the most common quinolone resistance-associated substitutions in *E. coli* (Ruiz, 2003), and their coexistence confers high-level fluoroquinolone resistance (Morgan-Linnell and Zechiedrich, 2007). Chromosomal genes (*mgrB*, *phoP*, *phoQ*, *pmrB*, and *pmrA*) involved in the susceptibility of bacteria to colistin were found to have no mutations, suggesting that colistin resistance of EC17GD31 is only related to the presence of *mcr-1*.

**TABLE 1:** Genomic features of *E. coli* EC17GD31.

**Chromosome/plasmid**	**Type**	**Size (bp)**	**GC content (%)**	**Resistance gene (s)**
EC17GD31	ST457	4,975,832	50.46	*bla*_CTX–M–__55_, *mdf(A)*
pGD31-MCR	IncI2	60,038	42.28	*mcr-1*
pGD31-NDM	IncA/C2	188,230	51.62	*aadA2*, *aadB*, *aphA6*, *bla*_NDM–1_, *bla*_OXA–__4_, *ble*_MBL_, *qacE*Δ*1*, *qnrA1*, *strA*, *strB*, *sul1*
pGD31-F25	IncFIB (pLF82)	110,686	45.70	-
pGD31-F1928	IncFIB (AP001918), IncFII, IncR	245,305	51.37	*aadA1*, *aadA2*, *aph(3*′*)-Ia*, *bla*_TEM–__1__B_, *cmlA1*, *dfrA12*, *floR*, Δ*mef(B)*, Δ*mig-14*, *qacH*, *sul2*, *sul3*, *tet(A)*, *tet(M)*

**TABLE 2:** Minimum inhibitory concentrations of tested antibiotic agents for *E. coli* EC17GD31, the transconjugant, and *E. coli* J53 recipient strain.

**Isolate**	**MIC (μg/ml)**																	
	**AMK**	**GEN**	**AMP**	**AMC**	**CTX**	**CAZ**	**CRO**	**FEP**	**ETP**	**IPM**	**MEM**	**FOX**	**CIP**	**CHL**	**CST**	**TET**	**TGC**	**SXT**
EC17GD31	128	>256	>256	32	>256	>256	>256	24	32	8	128	>256	128	256	4	256	0.06	>32
Transconjugant	2	1.5	6	4	0.06	1	0.03	0.16	0.008	1	0.015	8	0.03	8	4	2	0.06	0.47
*E. coli* J53	2	1.5	4	3	0.125	0.25	0.03	0.016	0.008	1	0.015	8	0.03	4	0.5	2	0.125	0.047

*AMK, amikacin; GEN, gentamicin; AMP, ampicillin; AMC, amoxicillin-clavulanic acid; CTX, cefotaxime; CAZ, ceftazidime; CRO, ceftriaxone; FEP, cefepime; ETP, ertapenem; IPM, imipenem; MEM, meropenem; FOX, cefoxitin; CIP, ciprofloxacin; CHL, chloramphenicol; CST, colistin; TET, tetracycline; TGC, tigecycline; SXT, trimethoprim-sulfamethoxazole.*

Multilocus sequence typing indicated that EC17GD31 belongs to ST457 according to the Warwick scheme. Several reports have described *E. coli* ST457 isolated from animals and hospitals also carries the *mcr-1* gene (Suzuki et al., 2016; Tada et al., 2017). Furthermore, this clone and ST410 were the first identified clones of clinical *E. coli* from Vietnam harboring *mcr-1* (Tada et al., 2017). Significantly, carbapenem resistance gene *bla*_NDM–1_ and colistin resistance gene *mcr-1* coexist in EC17GD31. To the best of our knowledge, this is the first report of a clinical *E. coli* strain from Vietnam that co-harbors *mcr-1* and *bla*_NDM–1_. NDM- and MCR-coproducing *Enterobacteriaceae* isolates, mainly *E. coli*, were reported in numerous other countries, e.g., China (Zheng et al., 2016), the United States (Mediavilla et al., 2016), the Arabian Peninsula (Sonnevend et al., 2016), Venezuela (Delgado-Blas et al., 2016), and Thailand (Paveenkittiporn et al., 2017). The NDM genes *bla*_NDM–1_ (Delgado-Blas et al., 2016; Zheng et al., 2016; Paveenkittiporn et al., 2017) and *bla*_NDM–__5_ (Mediavilla et al., 2016; Yang et al., 2016; Zheng B. et al., 2018), followed by *bla*_NDM–__9_ (Yao et al., 2016; Lai et al., 2017), were commonly detected in *E. coli* isolates co-carrying *bla*_NDM_ and *mcr-1*. The emergence and dissemination of NDM- and MCR-coproducing *Enterobacteriaceae* has become an urgent threat to clinical treatment and infection control.

### Genetic Features of Plasmid pGD31-MCR

**FIGURE 1:**
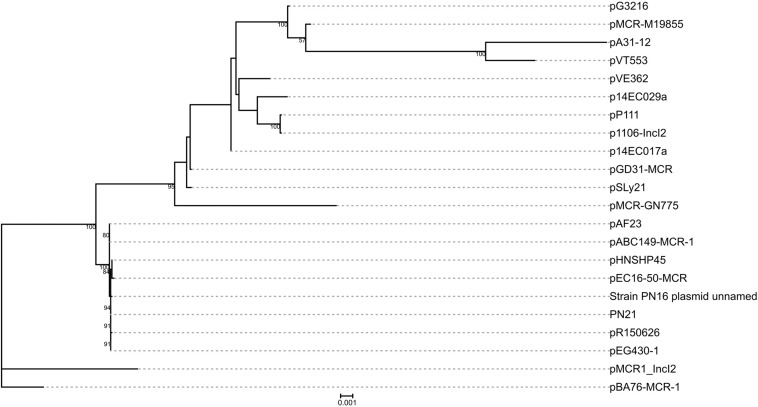
Phylogenetic analysis on pGD31-MCR and 21 other IncI2 *mcr-1*-carrying plasmids obtained from GenBank. The accession numbers of the plasmids are shown in Supplementary Table 1. Bootstrap values are shown next to nodes.

pGD31-MCR is a 60,038-bp circular plasmid bearing the *mcr-1* gene, with an average GC content of 42.28%. The plasmid belongs to the IncI2 group, which is the most prevalent MCR-1-producing plasmid group in Asia (Matamoros et al., 2017). pGD31-MCR only carries *mcr-1*, whereas some other IncI2-type plasmids harbor both the *mcr-1* and CTX-M ESBL genes (Sun et al., 2016; Matamoros et al., 2017). Colistin-resistant transconjugant indicated that the plasmid carrying the *mcr-1* gene was transferred to the *E. coli* J53 recipient strain (Table 2). This result showed that pGD31-MCR is a self-conjugative plasmid. Based on the core genome of IncI2 plasmids harboring *mcr-1*, the phylogenetic analysis revealed that pGD31-MCR shared the highest level of sequence similarity with pSLy21 (Genbank accession no. CP016405) in *E. coli* strain 210221272 isolated from a pig (Meinersmann et al., 2016; Figure 1). The sequence comparison of pGD31-MCR using BLASTn showed 99% identity and 97% coverage with pHNSHP45 (Genbank accession no. KP347127), the first identified plasmid harboring *mcr-1* (Liu et al., 2016). Similar to pHNSHP45, the *mcr-1* gene is located in a region with a structure of *nikB*-IS*Apl1*-*mcr-1*-*pap2*. The IS*Apl1*-mediated composite transposon was considered to be the primary genetic context of *mcr-1* (Snesrud et al., 2016; Wang et al., 2018a). Illegitimate recombination may results in the loss of IS*Apl1* flanking elements, leading to the stable existence of *mcr-1* in plasmids or chromosomes (Snesrud et al., 2016).

### A Novel Complex Class 1 Integron in Plasmid pGD31-NDM

**FIGURE 2:**
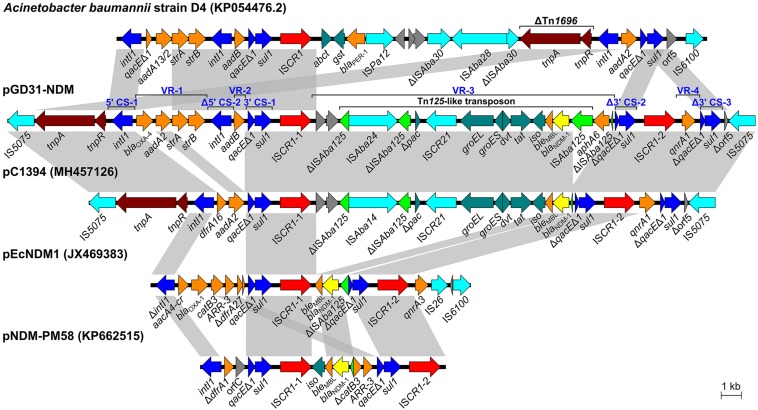
Schematic representation of the complex class 1 integron in pGD31-NDM and structural comparison with *Acinetobacter baumannii* strain D4, *Vibrio alginolyticus* plasmid pC1394, *E. coli* plasmid pEcNDM1, and *Proteus mirabilis* plasmid pNDM-PM58. Boxed arrows indicate the positions of open reading frames and their directions of transcription. Open reading frames encoding hypothetical proteins are portrayed by gray-shaded arrows. Gray-shaded areas denote more than 99% DNA identity between sequences. CS, conserved segment; VR, variable region.

Plasmid pGD31-NDM is a circular molecule of 188,230 bp with an average GC content of 51.37% that belongs to the IncA/C2 group. The plasmid harbors numerous antibiotic resistance genes, which together provide resistance to multiple classes of antibiotics, including aminoglycosides, β-lactams, quinolones, and sulfonamides (Table 1). Interestingly, all resistance genes of the plasmid are located in a 30,659-bp multidrug-resistant region with the structure of a complex class 1 integron consisting of two 5′ CSs, three 3′ CSs, two IS*CR1*s, and four VRs bearing resistance genes (Figure 2). The class 1 integron region contains a complete copy and a partial copy of the 5′ CS and a copy of the 3′ CS, similar to that of *Acinetobacter baumannii* strain D4 (Genbank accession no. KP054476.2) (Hamidian et al., 2015). The cassette regions of the class 1 integron in pGD31-NDM carry five resistance genes (*bla*_OXA–__4_, *aadA2*, *strA*, *strB*, and *aadB*), three of which were found in those of *A. baumannii* strain D4. The *bla*_OXA–__4_ gene encoding an ESBL is usually found in *Pseudomonas aeruginosa* (Marumo et al., 1999; Kainuma et al., 2018).

The IS*CR1*-1-linked VR-3 in pGD31-NDM contains two genes of unknown function, *aphA6* and a Tn*125* transposon carrying the *bla*_NDM–1_ gene (Figure 2). However, IS*Aba125* lying downstream of *bla*_NDM–1_ was interrupted by the insertion of IS*Aba24*. Interestingly, the IS*CR1*-1-associated VR in plasmid pC1394 (Genbank accession no. MH457126) is identical to that found in pGD31-NDM, except for the truncated IS*Aba125* lying downstream of *bla*_NDM–1_ and the lack of *aphA6*. IS*CR1* was found linked to various VRs carrying *bla*_NDM–1_ in *E. coli* plasmid pEcNDM1 (Genbank accession no. JX469383) and *Proteus mirabilis* plasmid pNDM-PM58 (Genbank accession no. KP662515). The construction of complex class 1 integrons harboring *bla*_NDM–1_ indicated that IS*CR1* is also an element responsible for the mobilization of the *bla*_NDM–1_ gene.

Similar to pECNDM1, pGD31-NDM harbors a second copy of IS*CR1* followed by quinolone-resistant gene *qnrA* (Figure 2). In pGD31-NDM, however, a duplicate of the 3′ CS of the class 1 integron is located immediately downstream of the *qnrA1* gene, revealing the presence of a complete structure of IS*CR1* containing *qnrA1*. The *ampR* gene, which is involved in the induction of class C β-lactamase, is usually found adjacent to *qnrA1* in IS*CR1*-linked regions (Rui et al., 2018), whereas it was missing in that of pGD31-NDM.

Unlike other plasmids, the complex class 1 integron in pGD31-NDM carries the class 1 integron region bearing two 5′ CSs and one 3′ CS and the two complete structures of IS*CR1*. The presence of these two complete structures of IS*CR1* may be the result of successive integration events following the model of Toleman et al. (2006). Free circular intermediates carrying the orf-orf-Tn*125*-like transposon-*aphA6*-Δ3′ CS-IS*CR1* element and *qnrA1*-Δ3′ CS-IS*CR1* element were generated via aberrant rolling-circle replications. These circular intermediates might then be rescued by homologous recombination via the 3′ CS of the class 1 integron. Thus, it would be possible for the complex integron to expand structure. Surprisingly, the overall form of the complex class 1 integron in pGD31-NDM is identical to that of pC1394 in *Vibrio alginolyticus* strain Vb1394 isolated from shrimp (Zheng Z. et al., 2018), except for the structure of the class 1 integron, the truncation of IS*Aba125*, and the existence of *aphA6* (Figure 2). Two copies of IS*5075* are adjacent to the boundaries of the complex integron, leading to putative genesis of an IS*5075*-mediated composite transposon, which may move the complex integron via transposition or homologous recombination. The formation of potential IS*5075*-mediated composite transposon raises a concern about the prevalence of this complex integron.

The linkage of the class 1 integron and IS*CR1* results in the extension of multidrug-resistant regions by adding resistance genes. In addition, the *mcr* and *bla*_NDM_ genes may be transferred to a same transconjugant, although they are located on various self-transmissible plasmids (Long et al., 2019). Altogether, IS*CR1*-mediated construction of complex class 1 integrons and co-transfer of *mcr* and *bla*_NDM_ play a significant role in forming extensive drug-resistant and pandrug-resistant bacteria, thus causing great challenges in the fight against antibiotic resistance.

### sul3-Type Integron in Plasmid pGD31-F1928

**FIGURE 3:**
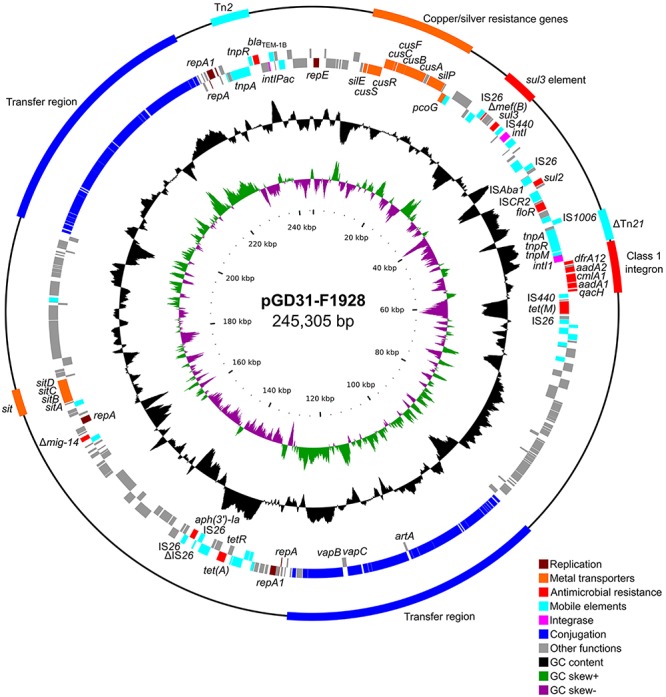
Circular representation of plasmid pGD31-F1928. The outermost circle indicates genetic elements, including resistance region, transposon, integron, and transfer region. The second and third circles show coding sequences on the forward and reverse strand of the plasmid, respectively. The fourth and fifth circles show the GC plot and GC skew graph, respectively. The innermost circle shows size in kb.

**FIGURE 4:**
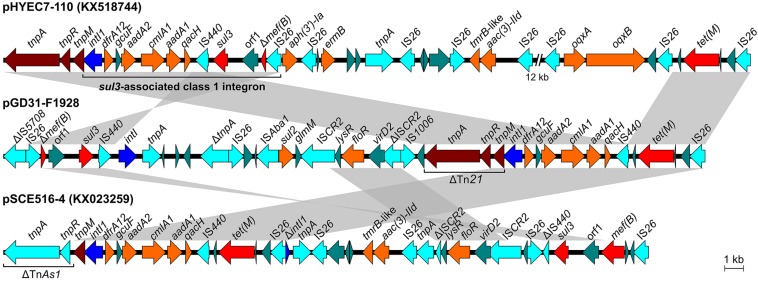
Structural comparison of regions harboring class 1 integron and *sul3* element of pGD31-F1928 and *E. coli* plasmid pSCE516-4 with *sul3*-associated class 1 integron of *E. coli* plasmid pHYEC7-110. Boxed arrows indicate the positions of open reading frames and their directions of transcription. Gray-shaded areas denote more than 99% DNA identity between sequences.

pGD31-F1928 is a 245,305-bp circular plasmid with an average GC content of 51.37% (Table 1). The plasmid harbors three replication systems (IncR, IncFIB, and IncFII), two conjugation regions, *sitABCD* operon, copper/silver resistance genes, Tn*2* transposon, a class 1 integron, and some resistance genes bounded by various insertion sequences (Figure 3). Antibiotic resistance genes are associated with resistance to aminoglycosides, β-lactams (penicillins and first-generation cephalosporins), chloramphenicol, florfenicol, trimethoprim, sulfonamides, and tetracyclines. The genetic context of sulfonamide resistance gene *sul2* and florfenicol resistance gene *floR*, *sul2*-*glmM*-IS*CR2*-*lysR*-*floR*-*virD2*-ΔIS*CR2*, was identified (Figure 4). This genetic environment is identical to that of plasmid pHNSHP45-2 (Genbank accession no. KU341381) and Tn*6450* transposon of *Proteus mirabilis* strain SNYG17 (Genbank accession no. MF805806) (Chen et al., 2018). The *sul2* and *floR* genes are usually found associated with IS*CR2*, the amino acid sequence of which is 65% similar to that of IS*CR1*, but IS*CR2* is not responsible for the formation of the complex class 1 integron (Toleman et al., 2006). In addition, pGD31-F1928 also carries a truncated *mig-14* gene, which relates to antimicrobial peptide resistance in *Salmonella enterica* (Brodsky et al., 2002) and *P. aeruginosa* (Jochumsen et al., 2011).

The *aadA1*, *aadA2*, *cmlA1*, *dfrA12*, and *qacH* genes are located in a gene cassette array of class 1 integron (Figure 4). The integron with a structure of *intI1*-*dfrA12*-*gcuF*-*aadA2*-*cmlA1*-*aadA1*-*qacH*-IS*440*-*sul3* is known as *sul3*-associated integron type I (Antunes et al., 2007). Surprisingly, only IS*440* was found downstream of the *qacH* gene, whereas *sul3* and a duplicate of IS*440* were located in another region of the plasmid (Figure 4). The IS*440*-*sul3* element lay upstream of an untyped integrase-encoding *intI* gene and linked to the region harboring a putative oxidoreductase gene (*orf1*) and *mef(B)* truncated by an IS*26* insertion. The linkage of *sul3* with *mef(B)*, a macrolide efflux gene, was first described in *E. coli* isolated from pigs (Liu et al., 2009). The *intI1*-*dfrA12*-*gcuF*-*aadA2*-*cmlA1*-*aadA1*-*qacH*-IS*440* region is adjacent to the *tetM*-IS*26* element, creating a new structure of a non-classic class 1 integron. This structure was also observed in plasmid pSCE516-4 (Genbank accession no. KX023259), but the integron is located upstream of the Tn*As1 tnpA*-*tnpR* element and Tn*21 tnpM* instead of the Tn*21 tnpA*-*tnpR*-*tnpM* element as in pGD31-F1928 and pHYEC7-110 (Genbank accession no. KX518744) (Figure 4). In addition, the genetic context of *sul3* in pSCE516-4 bears a truncated IS*440* and complete *mef(B)*, whereas pGD31-F1928 contains a complete IS*440* and truncated *mef(B)*. Altogether, we propose a hypothesis that the original class 1 integron in pGD31-F1928 is similar to the *sul3*-associated integron in pHYEC7-110. It is likely that the region containing *sul3*, *orf1*, and Δ*mef(B)* was flanked by IS*440* and IS*26*, leading to a rearrangement of the plasmid sequence promoted by the insertion sequences. As a result, *sul3* element, *sul3*-*orf1*-Δ*mef(B)-*IS*26*, was separated out of the integron, thus breaking the linkage between *sul3* and the class 1 integron. The connection of the class 1 integron and the *tet(M)*-IS*26* element may construct a putative *tet(M)*-associated class 1 integron.

## Conclusion

A multidrug-resistant *E. coli* strain EC17GD31 simultaneously harboring *mcr-1* and *bla*_NDM–1_ is identified, which alerts health officials to the presence of *mcr-* and *bla*_NDM_-positive *Enterobacteriaceae* in Vietnam. The close monitoring of MCR- and NDM-coproducing *Enterobacteriaceae* isolates is essential in order to safeguard the effectiveness of last-resort antibiotics. Additionally, this study revealed that the *bla*_NDM–1_ gene is located in a novel complex class 1 integron containing a class 1 integron region bearing two VRs and two complete structures of IS*CR1*. The characterization of the complex integron further demonstrates that IS*CR1* is a powerful genetic element that can mobilize a single gene as well as a composite transposon (e.g., Tn*125*-like transposon), therefore, the spread of resistance genes including *bla*_NDM–1_ can be anticipated. Although additional intermediates and progenitor plasmids were not found, the formation of the complex integron was most likely based on successive events of resistance gene mobilization. The findings of this study further support that complex interactions between antibiotic resistance genes and mobile elements play an important role in the creation and evolution of multidrug-resistant regions, thus facilitating bacteria to achieve either extensive drug resistance or pandrug resistance.

## Data Availability Statement

The raw data supporting the conclusions of this manuscript will be shared upon request submitted to the corresponding author.

## Ethics Statement

The studies involving human participants were reviewed and approved by the Institutional Ethics Committee in Biomedical Research, Pasteur Institute in Ho Chi Minh City, Vietnam. Written informed consent for participation was not required for this study in accordance with the national legislation and the institutional requirements.

## Author Contributions

VC and JJ conceived and designed the study. PT and LL performed the microbiological and molecular experiments. TI, SN, and DM performed the whole-genome sequencing experiments. H-NL-V, PT, and YM analyzed the data and prepared figures. H-NL-V wrote the manuscript with support from VC, PT, and LL. All authors contributed to manuscript revision, read, and approved the submitted version.

## Disclaimer

Material has been reviewed by the Walter Reed Army Institute of Research. There is no objection to its presentation and/or publication. The opinions or assertions contained herein are the private views of the author, and are not to be construed as official, or as reflecting true views of the Department of the Army or the Department of Defense. The investigators have adhered to the policies for protection of human subjects as prescribed in AR 70-25.

## Conflict of Interest

The authors declare that the research was conducted in the absence of any commercial or financial relationships that could be construed as a potential conflict of interest.

## Footnotes

1 http://www.eucast.org/clinical_breakpoints/

2 https://www.ncbi.nlm.nih.gov/genome/annotation_prok/

3 https://cge.cbs.dtu.dk/services/

4 https://www-is.biotoul.fr/

5 https://blast.ncbi.nlm.nih.gov/Blast.cgi
