# Supplementary material for: Complex Class 1 Integron in a Clinical Escherichia coli Strain From Vietnam Carrying Both mcr-1 and blaNDM–1
Source: Front Microbiol. 2019 Oct 31;10:2472. doi: 10.3389/fmicb.2019.02472 (PMC6834847; doi:10.3389/fmicb.2019.02472)
Supplement: Supplementary file 1 [file Table_1.DOCX]

**Supplementary data**

**Table 1.** Characteristics of *mcr-1*-positive IncI2 plasmids.

| No. | Plasmid | Host species | Origin | Accession number |
| --- | --- | --- | --- | --- |
| 1 | pVE362 | *Escherichia coli* | Food | AP018355 |
| 2 | pSLy21 | *Escherichia coli* | Animal | CP016405 |
| 3 | p14EC017a | *Escherichia coli* | Clinical sample | CP024135 |
| 4 | p14EC029a | *Escherichia coli* | Clinical sample | CP024142 |
| 5 | pGD31-MCR (this study) | *Escherichia coli* | Clinical sample | CP031296 |
| 6 | pHNSHP45 | *Escherichia coli* | Animal | KP347127 |
| 7 | pMCR1_IncI2 | *Escherichia coli* | Clinical sample | KU761326 |
| 8 | pVT553 | *Escherichia coli* | Animal | KU870627 |
| 9 | pABC149-MCR-1 | *Escherichia coli* | Clinical sample | KX013538 |
| 10 | pBA76-MCR-1 | *Escherichia coli* | Clinical sample | KX013540 |
| 11 | pAF23 | *Escherichia coli* | Clinical sample | KX032519 |
| 12 | pA31-12 | *Escherichia coli* | Animal | KX034083 |
| 13 | pP111 | *Salmonella enterica* subsp. *enterica* | Animal | KY120365 |
| 14 | pR150626 | *Salmonella enterica* subsp. *enterica* | Clinical sample | KY120366 |
| 15 | pMCR-GN775 | *Escherichia coli* | Clinical sample | KY471307 |
| 16 | pMCR-M19855 | *Escherichia coli* | Clinical sample | KY471315 |
| 17 | pEG430-1 | *Shigella sonnei* | Clinical sample | LT174530 |
| 18 | pG3216 | *Escherichia coli* | Clinical sample | MF693349 |
| 19 | Strain PN16 plasmid unnamed | *Escherichia coli* | Food | MG489944 |
| 20 | pEC16-50-MCR | *Escherichia coli* | Clinical sample | MG515249 |
| 21 | PN21 | *Escherichia coli* | Animal | MG557851 |
| 22 | p1106-IncI2 | *Escherichia coli* | Food | MG825374 |
